# New York Odontological Society

**Published:** 1904-06

**Authors:** 


### New York Odontological Society.

*(From Secretary's Report.)*

At the meeting of the New York Odontological Society, held at the Academy of Medicine, New York, Tuesday evening, April 19, 1904, Eustace H. Gane, Ph. C., read a paper entitled,

### DENTAL APPLICATIONS OF SOME NEW OXYGEN COMPOUNDS.

After a brief mention of the value of solutions of hydrogen dioxide to the dentist, the lecturer drew attention to a number of new compounds which readily liberated oxygen. The first of these to be introduced was sodium dioxide which was valuable because it had a saponifying action in addition to its germicidal properties. Its caustic properties and the danger in handling it were serious objections to its general use. Attention was then drawn to the fact that hydrogen dioxide would unite with certain saline substances, forming a number of interesting compounds, the most valuable of which are the compounds with carbonate of soda and borax. The first compound would do all that sodium dioxide would do, and did not have the disadvantages of the latter. The borax compound was milder in its action and useful where a slow oxidising action was required. These compounds were at present in the experimental stage, but promised to be of value to the physician as well as the dentist.

The author then pointed out that more and more attention was being paid to mouth hygiene. Dr. William Hunter had informed us that many disorders of the stomach may be attributed to the irritation caused by swallowed pus. This may happen not only in cases of pyorrhea alveolaris, but also from diseases of the gums, stomatitis due to septic plates, and to the bridge and crown work now so much in vogue. More dangerous still are pus organisms associated with dental caries; no pus organisms being so virulent as those grown in necrosed bone. Given the necessary conditions, diminished resistance on the part of the tissues, the pus organisms taken in by the mouth may be disease-producing.

The problem, then, of the oral therapeutist was to discover some means of keeping the mouth in a clean condition. Tooth powders heretofore manufactured were of little value, as no antiseptic could be introduced in sufficiently large amount to do the necessary work without injury to the soft tissues, while liquid dentifrices were weak in cleansing properties and chiefly served by producing a cooling effect, to give a false impression of cleanliness. Here was the point where the chemist could come to the dentist's and physician's aid. Among the new oxygen compounds was one which had been found available in the highest degree for the purposes of mouth sterilisation. This was the calcium dioxide which, thanks to the development of the electrical industries at Niagara, could now be produced on a commercial scale. This chemical added to a tooth powder base was found to give the powder properties hitherto thought impossible of attainment. In contact with the mouth fluids oxygen was liberated with the formation of milk of lime, an antacid as well as a germicidal action being thus attained. Moreover, in incipient dental caries where there were local acid areas this acid decomposed the calcium dioxide, liberating hydrogen dioxide at exactly the point where its germicidal properties were most needed.

By this means it was now possible to give to the patient a simple means not only of preserving the teeth from decay, but of preventing, to a large extent, the ingress of those pathogenic bacteria which frequently found a suitable field of development in the human mouth. An added advantage was the avoidance of the necessity of using a mouth wash as well as a tooth powder, something which is very difficult to induce the average person to do. The paper was illustrated with clinical experiments.

